# The Importance of Basic Sciences in Dental Education

**DOI:** 10.3390/dj12120382

**Published:** 2024-11-26

**Authors:** Anna Tostrup Kristensen, Noora Helene Thune, Qalbi Khan, Tor Paaske Utheim, Amer Sehic

**Affiliations:** 1 Institute of Oral Biology, Faculty of Dentistry, University of Oslo, 0316 Oslo, Norway; annatk@student.odont.uio.no (A.T.K.); nooraht@student.odont.uio.no (N.H.T.); qalbi.khan@odont.uio.no (Q.K.); utheim2@gmail.com (T.P.U.); 2 Department of Medical Biochemistry, Oslo University Hospital, 0424 Oslo, Norway; 3 Department of Plastic and Reconstructive Surgery, Oslo University Hospital, 0424 Oslo, Norway

**Keywords:** basic medicine, dentistry, genetics, innovation, precision medicine

## Abstract

The rapid advancements in biomedical sciences, including genomics, microbiome research, and bioinformatics, underscore the need for dental education to evolve to meet future challenges in public oral health and healthcare delivery. The integration of basic sciences into dental curricula is crucial to ensure that dental professionals are thoroughly prepared in these fundamental areas. Despite the widespread agreement on the necessity of including basic medical sciences in dental education, challenges such as curricular congestion, faculty economics, and infrastructural limits persist, complicating the integration of new scientific knowledge. Furthermore, there remains a significant lack of research concerning the optimal extent, timing, and focus of these subjects, whether biochemical, medical, or dental. Additionally, there is a need to address prevailing conceptions about the irrelevance of basic sciences to the field of dentistry, which necessitates a focus on teaching methodologies and pedagogical strategies. Therefore, it is essential to advance educational research that prepares future educators to integrate basic sciences into dental education through evidence-based teaching methods. The dental curriculum, which encompasses fundamental sciences, laboratory exercises, and clinical practice, must overcome considerable pedagogical challenges to effectively incorporate and balance these basic sciences within its educational structure.

## 1. Basic Sciences in Dental Education: Timing and Volume

In the current complex healthcare environment, effective collaboration among health service team members is essential for improving patient outcomes [1,2]. There is a broad international consensus that health professional students should be prepared for collaborative practice through interprofessional education [3]. International health organizations promote this approach to address challenges like an aging population, limited resources, and the need for better interdisciplinary teamwork [1,4,5]. Universities are encouraged to develop inclusive interprofessional education programs [4], making it essential for dental and medical students to learn cooperation early in their careers and to appreciate each profession’s unique contributions.

Most dental teaching institutions acknowledge that basic science is a fundamental element of the dental education program [6]. As the landscape of healthcare rapidly evolves, the complexity of cases that dentists encounter increases correspondingly [7]. Demographic changes, particularly an elderly population, have not only altered the common health challenges but also increased the need for a comprehensive understanding of systemic health [8]. Older patients often present with multiple comorbidities and are frequently on several medications, which also adds to the complexity of their dental care. Consequently, the treatment needs and risk factors of patients have advanced, highlighting the undeniable importance of basic medical knowledge in dental practices [9]. However, do we fully understand the extent of medical knowledge required by future dentists? This query leads to further contemplation about the optimal integration of medicine and basic science in dental education.

**Figure 1:**
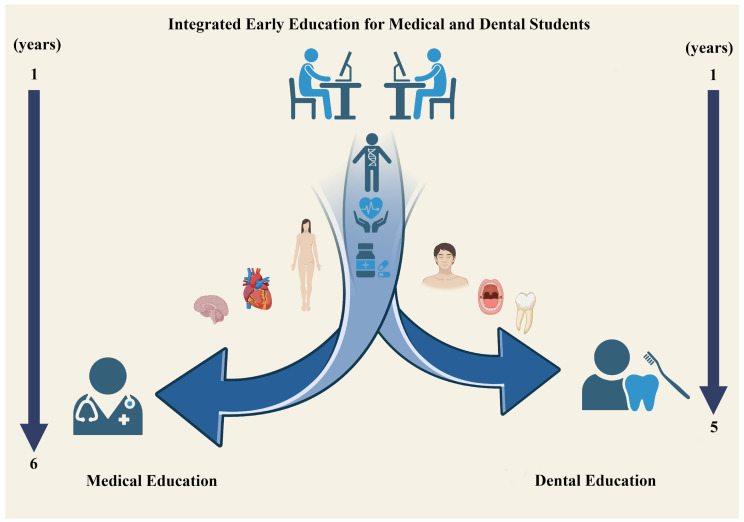
Integrating medical and dental education to establish an optimal pathway for developing future competent dental professionals presents significant challenges. In many dental schools, the curriculum is structured so that dental students undertake the same foundational medical courses as medical students during the first 1–2 years of their education. Both groups of students concentrate extensively on basic medical sciences before diverging into their respective fields of specialization—medicine and dentistry. The arrows in the figure represent the educational trajectory for both student groups, with numbers indicating the corresponding academic years. This figure illustrates the early interdisciplinary integration of the two programs and raises questions about whether this pattern is ideal.

In many dental schools, the curriculum is structured so that dental students complete the same foundational medical courses as medical students during the first two years of their education before moving on to specialized dental training (Figure 1). This period emphasizes basic medical sciences, covering subjects such as cell biology, genetics, anatomy, physiology, immunology, and pathology. Consequently, this foundational content is largely isolated from the clinical semesters, with minimal integration throughout the curriculum. This approach may also pose several other challenges, as previously observed and documented in the literature [10]. It could impact students’ professional identity by shifting their focus away from their original vocational goals. Therefore, this structure might prompt both medical and dental students to reconsider their educational paths, potentially choosing alternative fields of study instead of their original program. Such changes could also increase feelings of ambiguity about professional identity among remaining students who may perceive their choices as less valuable or inferior. Therefore, it is essential for dental students to maintain a clear understanding of their importance and role within the profession, ensuring their future success and stability. Consequently, for this interdisciplinary approach to be most effective, it is essential to consider the methods, timing, and volume of teaching these subjects.

A key component of dental education should, therefore, be the ability to incorporate basic medical knowledge into clinical practice. Given that these courses are primarily concentrated during the first two years and not extended throughout the subsequent clinical years, it remains uncertain whether students can adequately recall and apply necessary biomedical concepts that they learned at the beginning of their studies. Could this educational model carry consequences for future dental practitioners? This raises several crucial questions: How much medical knowledge is essential for dental practitioners? When should this knowledge be taught to ensure it is effectively retained and applied in clinical practice? And, more importantly, how can we incorporate this knowledge in a manner that demonstrates its practical utility? Can existing dental curricula effectively address these challenges, or is it necessary to reconsider our approach to dental education?

## 2. The Importance of Biology and Medicine in Dentistry

A simple example of the connection between complex biology and dentistry is the tooth, which serves as a valuable experimental model for studying fundamental mechanisms of organ development, including morphogenesis, cell interactions, cell differentiation, and the production and mineralization of extracellular matrices [11,12]. Furthermore, to engage in bone regeneration research, it is essential to understand what osteoblasts are and what they do [13]. New biomaterials require a deep understanding of physics and biochemistry [14], whereas comprehensive registration of erosive lesions on dental enamel and their depths in small teeth such as mouse molars are best understood through scanning electron microscopy [15]. These are just a few examples illustrating the clear links between intricated biology and dentistry.

On the other hand, oral diseases are increasingly associated with prevalent noncommunicable diseases, including cardiovascular diseases, diabetes, and cancers [16]. These conditions share common risk factors, such as social determinants (e.g., low income, low education levels) and behaviors (e.g., frequent smoking, high sugar intake). Evidence also highlights associations between poor oral health and other conditions, such as respiratory diseases, sleep-related breathing disorders, and cognitive decline [16]. This emphasizes the importance of the recent WHO call for strengthened cross-sectoral collaboration between general and oral health. The links between poor oral health and general health conditions highlight the need for essential, evidence-based, bidirectional expertise and skill sharing between medical doctors and dentists. In this context, increased focus is being given to the role of medical doctors in performing oral health assessments, particularly for patients with limited access to routine oral healthcare [17]. Dentists recognize the importance of medical screening, particularly for cardiovascular diseases, diabetes, and infectious diseases, including hepatitis and HIV [18]. Nonetheless, medical emergencies still frequently occur in dental practices, encompassing events such as vasovagal syncope, angina, hypoglycemia, epileptic seizures, choking, asthma, anaphylaxis, and cardiac arrest [19]. Consequently, maintaining an updated medical history, as supported by previous studies, is essential for significantly reducing the risk of such emergencies in dental practice [20].

## 3. Advancing Basic Science in Dental Education Through Vertical Integration

Integrating basic science into clinical instruction poses a substantial challenge for many dental schools, as the basic science curriculum in the first two years is typically structured as discrete courses managed by individual departments [21]. Nonetheless, early clinical engagements have been linked to enhanced comprehension of basic science’s relevance, improvement in attitudes, and accelerated development of clinical competencies [22,23]. Additionally, students benefit from the early integration of basic science, which helps develop a cohesive understanding of the relationship between pathological features and clinical diagnoses, enhancing retention and the application of knowledge in clinical settings [24]. Furthermore, such initial clinical experiences can rekindle students’ original passion for dentistry, particularly valuable when they begin to experience fatigue due to extensive theoretical coursework. Consequently, prioritizing increased patient interaction in the first year of dental education is recommended for dental schools to better support student learning and engagement [25].

Vertical integration combines basic and clinical sciences to bridge the traditional gap between preclinical and clinical phases, maintaining a focus on basic sciences throughout the undergraduate years [26]. These integrative strategies have not only improved students’ attitudes towards basic sciences [27] but have also fostered deeper and more comprehensive learning [28], enhancing their grasp of fundamental scientific principles. The significance of vertical integration may be exemplified by the application of essential genetic knowledge in clinical dentistry practices. Genetic predispositions have been linked to a variety of oral health conditions, such as periodontal diseases [29], as well as congenital dental anomalies, like tooth agenesis [30]. These genetic factors not only influence the composition of the oral microbiome but also affect patient behaviors, including the prevalence of dental anxiety [31]. Today, we also know that individual susceptibility to dental erosion and caries appears to be influenced by variations in the oral environment and dental enamel [32,33]. Specific genes that code for enamel matrix proteins such as amelogenin, enamelin, tuftelin, and tuftelin interaction protein 11 are linked to an increased vulnerability to both dental erosion and caries [33]. There has been significant advancement and interest in pinpointing genetic determinants that contribute to the onset of erosion and caries, and despite over a century of research into their pathogenesis, these conditions remain widespread globally. Consequently, there is a pressing need for new approaches to shield individuals who are at elevated risk. Therefore, vertical integration and the understanding of these genetic influences are critical for developing personalized dental care strategies that consider individual genetic profiles, ultimately enhancing clinical outcomes [34].

Collectively, a profound grasp of basic science enhances diagnostic accuracy and is heavily relied upon by expert clinicians facing complex cases [35]. The collaboration between basic scientists and clinicians, though intended to be synergistic, frequently reveals disconnects that can foster misconceptions about the relevance of basic science to clinical practice [36]. Despite these efforts, significant transformative changes in the curriculum have often been elusive, indicating a need for ongoing evaluation and adaptation of educational strategies to better integrate basic science with clinical practice effectively [36]. Furthermore, when curriculum changes are considered, a key question arises: if the program duration remains unchanged, what content must be removed? This is a significant challenge that must be carefully addressed in any curriculum revision.

## 4. Conclusions and Future Perspectives

Effective integration of medical and basic sciences throughout both the preclinical and clinical phases of dental education is required. Improved knowledge retention and curriculum integration offer clear benefits for the clinical practice of students and future dentists. For instance, teaching blood and immunology alongside oral medicine enhances comprehension, while linking caries development with microbiology provides a deeper understanding of disease mechanisms. Similarly, studying genetics in conjunction with both inherited and acquired dental malformations facilitates a more comprehensive approach to dental pathologies. Moreover, familiarity with AI and data ethics, particularly in applications like genetic diagnostics, can equip dental students with essential interdisciplinary skills for the future [5].

The dental curriculum, densely packed with fundamental sciences, laboratory exercises, and clinical practice, faces significant obstacles in effectively integrating and balancing basic sciences into its educational framework. The conventional segregation between basic sciences and clinical training further impedes a unified educational strategy. Despite the consensus on the necessity of including basic sciences in dental education, research remains scant regarding the optimal extent, timing (in terms of preclinical course, year, or semester), and specific focus (whether biochemical, medical, or dental) that would best serve the dental profession.

Additionally, addressing misconceptions about the irrelevance of basic sciences to dentistry necessitates an emphasis on teaching methodologies and pedagogical strategies. Therefore, it is crucial to pursue educational research that provides future educators with an evidence-based approach to teaching. Furthermore, to the best of our knowledge, the retention of basic science knowledge in dental education and practice has not been thoroughly studied. This area warrants further investigation, as it could provide valuable insights to optimize both the timing and volume of basic science instruction. This applies to dental-specific sciences, such as craniofacial anatomy and physiology, as well as broader medical sciences, including genetics, microbiology, immunology, and pathology.

## Data Availability

The original contributions presented in the study are included in the article; further inquiries can be directed to the corresponding author.
